# Development of an Individualized Ubiquitin Prognostic Signature for Clear Cell Renal Cell Carcinoma

**DOI:** 10.3389/fcell.2021.684643

**Published:** 2021-06-22

**Authors:** Yue Wu, Xi Zhang, Xian Wei, Huan Feng, Bintao Hu, Zhiyao Deng, Bo Liu, Yang Luan, Yajun Ruan, Xiaming Liu, Zhuo Liu, Jihong Liu, Tao Wang

**Affiliations:** ^1^Department of Urology, Tongji Hospital, Tongji Medical College, Huazhong University of Science and Technology, Wuhan, China; ^2^Institute of Urology, Tongji Hospital, Tongji Medical College, Huazhong University of Science and Technology, Wuhan, China; ^3^School of Health Sciences, Wuhan University, Wuhan, China; ^4^Department of Oncology, Tongji Hospital, Tongji Medical College, Huazhong University of Science and Technology, Wuhan, China

**Keywords:** clear cell renal cell carcinoma, ubiquitin, prognostic signature, prognosis, bioinformatics

## Abstract

Clear cell renal cell carcinoma (ccRCC) is a common tumor type in genitourinary system and has a poor prognosis. Ubiquitin dependent modification systems have been reported in a variety of malignancies and have influenced tumor genesis and progression. However, the molecular characteristics and prognostic value of ubiquitin in ccRCC have not been systematically reported. In our study, 204 differentially expressed ubiquitin related genes (URGs) were identified from The Cancer Genome Atlas (TCGA) cohort, including 141 up-regulated and 63 down-regulated URGs. A total of seven prognostic related URGs (CDCA3, CHFR, CORO6, RNF175, TRIM72, VAV3, and WDR72) were identified by Cox regression analysis of differential URGs and used to construct a prognostic signature. Kaplan-Meier analysis confirmed that high-risk patients had a worse prognosis (*P* = 1.11e-16), and the predicted area under the receiver operating characteristic (ROC) curves were 0.735 at 1 year, 0.702 at 3 years, and 0.744 at 5 years, showing good prediction accuracy. Stratified analysis showed that the URGs-based prognostic signature could be used to evaluate tumor progression in ccRCC. Further analysis confirmed that the signature is an independent prognostic factor related to the prognosis of ccRCC patients, which may help to reveal the molecular mechanism of ccRCC and provide potential diagnostic and prognostic markers for ccRCC.

## Introduction

Renal cell carcinoma (RCC) is one of the most aggressive genitourinary tumors, accounting for about 4% of adult malignancies (Zhai et al., 2019). According to statistics, 76,080 new kidney cancer cases and 13,780 kidney cancer deaths are expected to occur in the United States in 2021 (Siegel et al., 2021). Clear cell renal cell carcinoma (ccRCC) is the most studied and common subtype of RCC, accounting for approximately 80% of all RCC (Escudier et al., 2019). CcRCC is a malignant and substantial tumor originating from proximal renal tubular epithelial cells, with high metastasis rate and poor prognosis. The 5-year survival rate for advanced ccRCCs is only 11.7% (Siegel et al., 2017). About 30% of patients with metastatic ccRCC at the time of initial diagnosis, and approximately 30% of patients relapse after complete removal of the primary tumor (Motzer et al., 2008; Nerich et al., 2014). Thus, a comprehensive understanding of the pathogenesis of ccRCC, identification of biomarkers, and development of effective early screening and diagnosis methods are of great significance for prognosis prediction and treatment of ccRCC.

Post-translational modification (PTM) is a covalent change that occurs during or after translation of almost all proteins. PTM induces covalent linkage between proteins and functional groups including phosphate, acetyl, methyl and ubiquitin through a variety of signaling pathways, thereby regulating the localization, stability, activity, interaction or folding of proteins, thus influencing various biological processes (Deribe et al., 2010; Chiang and Gack, 2017). Among PTM types, ubiquitin dependent modification system is one of the major PTM systems (Hershko and Ciechanover, 1998). Ubiquitin is a highly conserved protein containing 76 amino acids. Ubiquitin modified proteins are catalyzed by three enzyme cascades consisting of ubiquitin-activating enzymes (E1s), ubiquitin-conjugating enzymes (E2s), and ubiquitin protein ligases (E3s) (Pickart, 2001). In this process, the ubiquitin-binding domain-containing protein (UBD) (Husnjak and Dikic, 2012), proteins containing ubiquitin-like domains (ULDs) (Upadhya and Hegde, 2003), and deubiquitinases (DUBs) (Nijman et al., 2005) play a negative regulatory role.

Studies have shown that dysregulation of the ubiquitin dependent modification system plays a key role in many diseases, including neurodegenerative diseases, autoimmune diseases, and malignancies (Seeler and Dejean, 2017; Rape, 2018). Lipkowitz and Weissman (2011) found that mutations or dysregulation of E3s expression are associated with poorer survival and prognosis in a variety of cancers. Another study reported that maternally expression gene 3 (Meg3) and miR-3163 may synergically inhibit Skp2 translation in non-small cell lung cancer cells, thereby inhibiting cancer cell growth (Su et al., 2016). In the field of RCC, Zhang et al. (2020) found that the ubiquitin ligase KLHL2 inhibited the progression of RCC by promoting the degradation and ubiquitination of ARHGEF7 protein. Other studies have shown that low ubiquitin-specific protease 2 mRNA expression is associated with poor prognosis of ccRCC, which has prognostic and diagnostic value (Meng et al., 2020). However, most of the current functional studies have only focused on single genes, few studies have systematically explored the molecular characteristics and prognostic potential of ubiquitin related genes (URGs) in ccRCC using high-throughput sequencing expression profile datasets. Therefore, in this study, we systematically explored the molecular characteristics and prognostic potential of these URGs in ccRCC, and preliminarily revealed the complex biological functions and immune processes involved in these molecules as well as their regulatory networks.

## Materials and Methods

### Data Download and Differential Expression URGs Analysis

Transcriptome data (read counts) containing 72 normal renal tissue samples and 539 ccRCC samples, together with corresponding clinical information, were downloaded from The Cancer Genome Atlas (TCGA)^1^ database. Then, 27 E1s, 109 E2s, 1153 E3s, 164 DUBs, 396 UBDs, and 183 ULDs were collected from the iUUCD 2.0 database^2^ (Gao et al., 2013), and 1,367 URGs were identified after duplication removal, and extracted 1,234 ccRCC-related URGs. Subsequently, the read counts data was preprocessed by ‘‘edgeR’’ package^3^, including deleting the genes whose average expression was less than 1 and normalizing the expression data with the trimmed mean of M-values algorithm. | log2 fold change (FC)| > 1.0 and false discovery rate (FDR) < 0.05 were considered to be differently expressed URGs. Additionally, the E-MTAB-1980 cohort was obtained from the ArrayExpress database^4^ as an external validation cohort. The microarray data were background adjusted and normalized using robust multi-array analysis (RMA) method in “Affy” package.

### Construction and Assessment of URGs Associated Prognostic Signature

To screen out prognostic related URGs, we first determined the association between differentially expressed URG expression levels and overall survival (OS) in ccRCC patients by univariate Cox regression analysis, and significant URGs associated with OS was determined when *P*-value was less than 0.05. Next, the least absolute shrinkage and selection operator (LASSO) Cox regression analysis was performed on these preliminary screened URGs using the “glmnet” package to identify the valuable prognostic URGs. Finally, we further screened the URGs most associated with prognosis through multivariate Cox proportional hazards regression analysis. We then constructed a prognostic signature based on the β coefficients of multivariate Cox regression analysis and the expression values of corresponding URGs. The risk score was calculated according to the following formula:

*Risk score* = $\sum_{i\,1}^{n}E\,x\,p\,i\,\beta \,i$,

in the above formula, Exp and β represent gene expression level and regression coefficient, respectively. Subsequently, Patients with ccRCC in the TCGA cohort were grouped according to the median risk score. Kaplan-Meier analysis was used to compare the difference in OS between high- and low-risk groups. Next, we constructed receiver operating characteristic (ROC) curves based on the “Survival ROC” package to explore the predictive power of the URGs-based risk signature. Moreover, we divided the whole TCGA cohort into two subsets as internal validation cohorts and the E-MTAB-1980 cohort as an external validation cohort to verify the prediction performance and stability of the URGs-based prognostic signature, respectively.

### Correlation Between Prognostic Signature, Prognostic URGs, and Clinical Characteristics

To explore the clinical value of the URGs-based prognostic signature, Kaplan-Meier analysis was conducted to investigate the differences in prognosis of ccRCC patients under different clinical characteristics stratification. We also compared the differences of risk score for different clinical characteristics to explore whether prognostic signature could assess the degree of tumor progression. Moreover, we stratified the expression levels of these URGs by different clinical characteristics and compared the differences in their expression levels to preliminarily reveal the possible roles of these URGs in ccRCC.

### Multidimensional Regulatory Network of Prognostic URGs and Functional Enrichment Analysis

We downloaded transcription factors (TFs) associated with tumorigenesis and progression from the Cistrome Project^5^, extracted ccRCC-related TFs and obtained differentially expressed TFs from the TCGA cohort. Then, we performed the co-expression of differentially expressed TFs and prognostic URGs to explore their regulatory relationship based on the criteria of | Cor| > 0.3 and P < 0.001. Next, we performed gene ontology (GO) and Kyoto Encyclopedia of Genes and Genomes database (KEGG) function enrichment analysis on these differentially expressed URGs. The biological functions and molecular mechanisms of these URGs were revealed through GO annotation, including biological processes, cell components and molecular functions, and the key signal regulatory pathways of URGs were revealed through KEGG enrichment analysis. These analyses were performed using the ‘‘clusterProfiler’’^6^package.

### Relationship Between Prognostic Signature and Degree of Immune Cell Infiltration

Since the ubiquitin dependent modification system is thought to profoundly influence the maturation of immune cells and shape the tumor microenvironment (Zhu et al., 2020), we evaluated the differences in the degree of immune cell infiltration between different subgroups based on the cell type identification by estimating relative subsets of RNA transcripts (CIBERSORT) algorithm. CIBERSORT is a deconvolution algorithm developed by Newman et al. (2015) that evaluates the relative abundance of immune cell infiltration in each patient based on data from 22 sets of genes associated with the infiltration of immune cells. The CIBERSORT algorithm was simulated 1,000 times, and the results were obtained according to *P* < 0.05.

### Evaluation of the Prognostic Significance of Different Clinical Characteristics in ccRCC Patients and Construction of a Nomogram

We then performed univariate and multivariate Cox regression analysis for each clinical characteristic and risk score to assess its clinical prognostic significance. Subsequently, we used the “rms” package to construct a nomogram combining different clinical characteristics and risk score to establish a quantitative prediction method for prognosis of ccRCC patients. Next, the calibration curves at different time points were plotted to evaluate the performance of the nomogram. Moreover, we further evaluated the predictive performance of the nomogram using the TCGA and E-MTAB-1980 cohorts.

### Immunohistochemical (IHC) Staining Analysis

To further verify the protein expression of these prognostic URGs, we used IHC staining assay to detect the expression levels of these genes in paraffin-embedded tissues of ccRCC and adjacent non-tumor renal tissues. The paraffin embedded tissue was stained in 5 μm continuous sections. The specific procedures for paraffin section immunohistochemistry of kidney tissue are described above (Li et al., 2010). IHC assayed against CDCA3, CHFR, TRIM72, VAV3, and WDR72. Primary antibodies against CDCA3, CHFR, VAV3, and WDR72 were purchased from ABclonal (Wuhan, China). Primary antibodies against TRIM72 were purchased from Bioss (Beijing, China). All experiments were conducted independently for at least three times. The images were observed and obtained with the Pannoramic SCAN (3DHISTECH, Hungary). Image Pro Plus software was used to analyze and quantify the IHC results.

## Results

### Analysis of Differentially Expressed URGs in ccRCC

**FIGURE 1:**
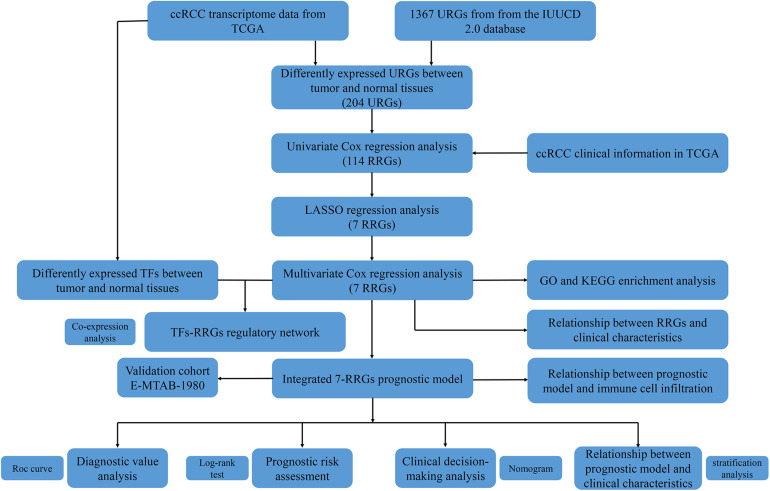
Flowchart for developing an individualized ubiquitin-based prognostic signature for clear cell renal cell carcinoma (ccRCC). We developed the URGs-based prognostic signature using the TCGA cohort and validated it in the ArrayExpress cohort.

**FIGURE 2:**
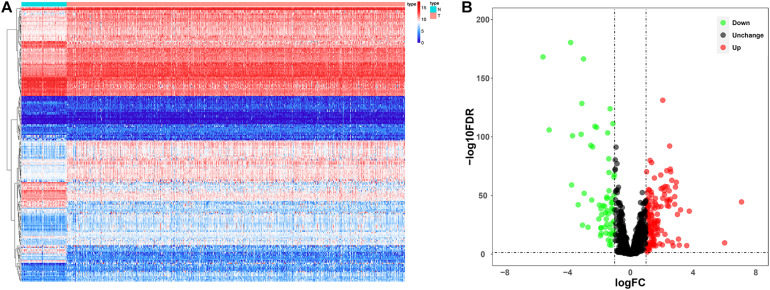
Expression and distribution of differentially expressed URGs in ccRCC. **(A)** The differential expression of 204 ubiquitin related genes (URGs) in ccRCC tissue samples (*n* = 539) compared with normal renal samples (*n* = 72) is shown in the volcano plot. The red plot represented up-regulated URGs, the green plot represented down-regulated URGs; **(B)** the differential expression of 204 URGs in ccRCC tissue samples (*n* = 539) compared with normal renal samples (*n* = 72) is shown in the heatmap (the statistical method was multiple hypothesis testing).

Since the molecular characteristics associated with ubiquitin and their prognostic potential in ccRCC are still unclear, we comprehensively explored the key role and clinical significance of URGs in ccRCC. Figure 1 shows the research roadmap. We first obtained RNA sequencing data from the TCGA database containing 72 normal renal tissue samples and 539 ccRCC samples. Subsequently, according to the | log_2_ FC| > 1.0 and FDR < 0.05, a total of 204 differentially expressed URGs were identified, of which 141 were up-regulated and 63 were down-regulated. The expression distribution of these URGs is shown in Figures 2A,B.

### Construction and Assessment of URGs-Based Prognostic Signature

For these differentially expressed URGs, we first identified 114 prognostic URGs by univariate Cox proportional hazards regression analysis (Supplementary Table 1). Then, LASSO regression analysis further screened out seven URGs, including *CDCA3*, *CHFR*, *CORO6*, *RNF175*, *TRIM72*, *VAV3*, and *WDR72*. The trajectory changes of these independent variable coefficients are shown in Supplementary Figure 1A, and the Supplementary Figure 1B shows the model construction using cross validation. We then performed multivariate Cox proportional hazards regression analysis on these seven URGs and finally identified the seven URGs most associated with prognosis, including *CDCA3*, *CHFR*, *CORO6*, *RNF175*, *TRIM72*, *VAV3*, and *WDR72*. Finally, we used the β coefficients of multivariate Cox proportional hazards regression analysis to establish a prognostic signature (Table 1), and multiplied these coefficients by the expression level of each URG to obtain the risk score. The risk score was calculated according to the following formula:

**TABLE 1:** Multivariate Cox regression analysis to identify prognosis-related URGs.

**Gene**	**Coef**	**Exp(coef)**	**se(coef)**	**z**	**Pr(> | z|)**
CDCA3	0.1726	1.1884	0.1072	1.6106	0.1073
CHFR	0.0788	1.0820	0.2199	0.3583	0.7202
CORO6	0.0898	1.0939	0.0613	1.4648	0.1430
RNF175	0.1389	1.1490	0.0755	1.8398	0.0658
TRIM72	0.0897	1.0939	0.0543	1.6535	0.0982
VAV3	−0.1586	0.8533	0.0822	−1.9291	0.0537
WDR72	−0.1202	0.8868	0.0480	−2.5015	0.0124

*Coef, coefficient. The statistical method was multiple hypothesis testing.*

Risk score = (0.1726 × Exp CDCA3) + (0.0788 × Exp CHFR) + (0.0898 × Exp CORO6) + (0.1389 × Exp RNF175) + (0.0897 × Exp TRIM72) + (−0.1586 × Exp VAV3) + (−0.1202 × Exp WDR72).

**FIGURE 3:**
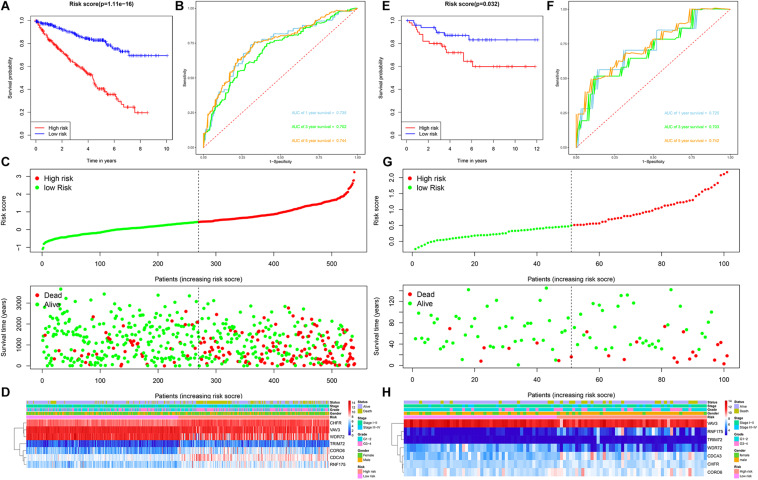
Prognostic signature analysis of ccRCC patients in the TCGA and E-MTAB-1980 cohorts. **(A)** Kaplan-Meier survival curve analysis in the high-risk and low-risk ccRCC patients in the TCGA cohort, patients in the entire TCGA cohort were divided into low-risk (*n* = 270) and high-risk (*n* = 269) groups based on the median risk score; **(B)** time-dependent ROC curves show area under curve values at 1-, 3-, and 5-year in the TCGA cohort ccRCC patients (*n* = 539); **(C)** risk score and survival status distribution of ccRCC patients in the TCGA cohort (*n* = 539); **(D)** heatmap of prognostic URGs expression under different parameters in the TCGA cohort ccRCC patients (*n* = 539); **(E)** Kaplan-Meier survival curve analysis in the high-risk and low-risk ccRCC patients in the E-MTAB-1980 cohort (*n* = 101), patients in the E-MTAB-1980 cohort were divided into low-risk (*n* = 51) and high-risk (*n* = 50) groups based on the median risk score; **(F)** time-dependent ROC curves show area under curve values at 1-, 3-, and 5-year in the E-MTAB-1980 cohort ccRCC patients (*n* = 101); **(G)** risk score and survival status distribution of ccRCC patients in the E-MTAB-1980 cohort (*n* = 101); **(H)** heatmap of prognostic URGs expression under different parameters in the E-MTAB-1980 cohort ccRCC patients (*n* = 101) (the statistical method was a log-rank test for a single factor).

**FIGURE 4:**
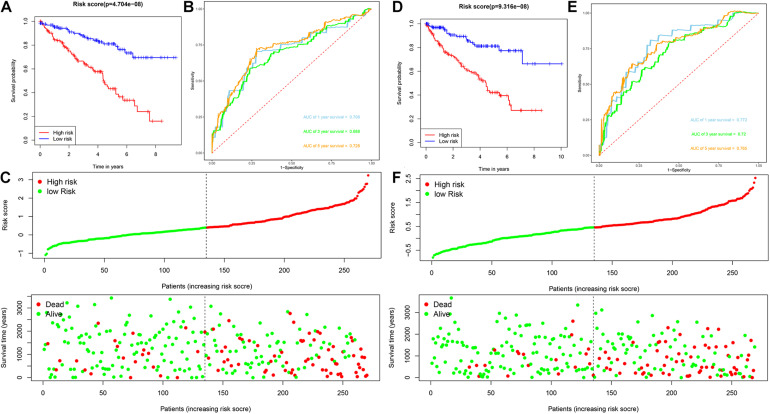
Analysis of prognostic signature of ccRCC patients in the training and test subsets based on the TCGA cohort. **(A)** Kaplan-Meier survival curve analysis in the high-risk and low-risk ccRCC patients in the training subset, patients in the training subset were divided into low-risk (*n* = 135) and high-risk (*n* = 135) groups based on the median risk score; **(B)** time-dependent ROC curves show area under curve values at 1-, 3-, and 5-year in the training subset ccRCC patients (*n* = 270); **(C)** risk score and survival status distribution of ccRCC patients in the training subset (*n* = 270); **(D)** Kaplan-Meier survival curve analysis in the high-risk (*n* = 134) and low-risk (*n* = 135) ccRCC patients in the test subset, patients in the test subset were divided into low-risk (*n* = 135) and high-risk (*n* = 134) groups based on the median risk score; **(E)** time-dependent ROC curves show area under curve values at 1-, 3-, and 5-year in the test subset ccRCC patients (*n* = 269); **(F)** risk score and survival status distribution of ccRCC patients in the test subset (*n* = 269).

Patients with ccRCC in the TCGA cohort were grouped according to the median risk score. Survival analysis by Kaplan-Meier method showed that patients in the high-risk group had a shorter OS than those in the low-risk group (*P* = 1.11e-16, Figure 3A), suggesting that the signature could accurately distinguish between ccRCC patients with poor prognosis. We then evaluated the predictive power and accuracy of the URGs-based risk signature according to ROC curve analysis, and the predicted area under the ROC curves (AUC) were 0.735 at 1 year, 0.702 at 3 years, and 0.744 at 5 years (Figure 3B). The risk score and survival status distribution of each patient are shown in Figure 3C, suggesting that a higher risk score is associated with a higher mortality rate of ccRCC patients. Figure 3D shows the expression heatmap assessed by clinical characteristics and risk score. Additionally, we used the E-MTAB-1980 cohort as an external cohort to further evaluate whether the prognostic signature has similar predictive performance and accuracy in other ccRCC patient cohorts. Similarly, Survival analysis by Kaplan-Meier method showed a worse prognosis for patients in the high-risk group (*P* = 0.032, Figure 3E). The predicted AUCs were 0.725 at 1 year, 0.703 at 3 years, and 0.742 at 5 years (Figure 3F). The risk score and survival status distribution of each patient are shown in Figure 3G, and Figure 3H shows a heatmap of expression in the E-MTAB-1980 cohort, based on clinical characteristics and risk score. Moreover, to further verify the prognostic signature, we divided the TCGA cohort into two similar subsets (training, *n* = 270; test, *n* = 269) for signature validation, respectively. In the training subset, survival analysis showed that patients in the high-risk group had a worse prognosis (*P* = 4.704e-08, Figure 4A). The predicted AUCs were 0.706 at 1 year, 0.688 at 3 years, and 0.728 at 5 years (Figure 4B). The risk score and survival status distribution of each patient are shown in Figure 4C. Analysis of the test subset shows similar results (Figures 4D–F). Therefore, we have a reason to believe that the URGs-based prognostic signature has good prediction performance and stability.

### Prognostic Significance of the Signature Under Different Clinical Characteristics Stratification

**FIGURE 5:**
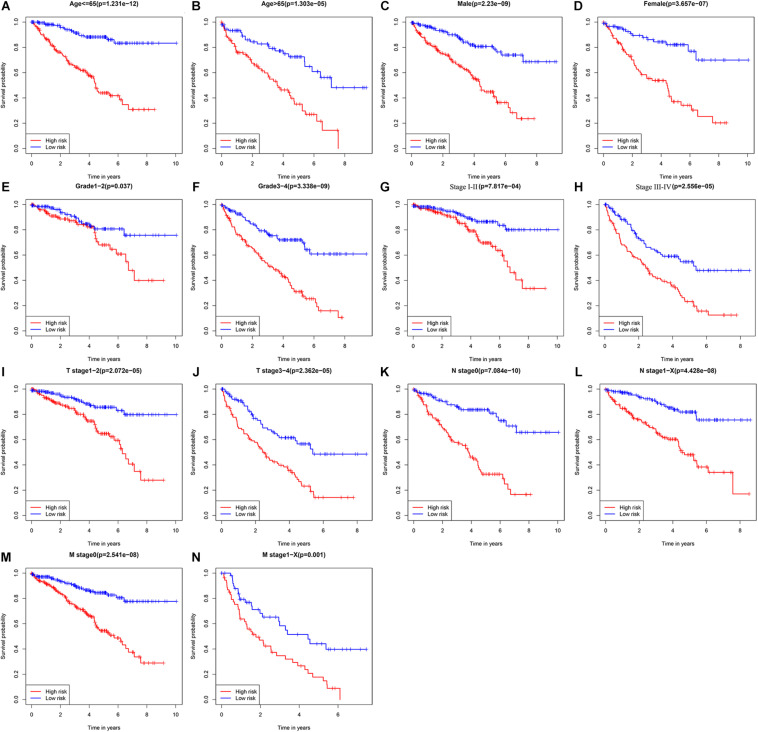
Kaplan-Meier survival curves analysis stratified by different clinical parameters. **(A)** Age ≤ 65 (*n* = 353); **(B)** age > 65 (*n* = 186); **(C)** male (*n* = 353); **(D)** female (*n* = 186); **(E)** grade 1–2 (*n* = 249); **(F)** grade 3–4 (*n* = 282); **(G)** stage I–II (*n* = 331); **(H)** stage III–IV (*n* = 205); **(I)** T stage 1–2 (*n* = 349); **(J)** T stage 3–4 (*n* = 190); **(K)** N stage 0 (*n* = 241); **(L)** N stage 1-X (*n* = 298); **(M)** M stage 0 (*n* = 428); **(N)** M stage1-X (*n* = 109) (the statistical method was a log-rank test for a single factor).

To explore the clinical value of the URGs-based prognostic signature, ccRCC patients in the TCGA cohort were stratified according to different clinical characteristics (including age, gender, tumor grade, tumor stage, T stage, N stage, and M stage). Survival analysis was performed by Kaplan-Meier method, and the results showed that the prognosis of patients in each high-risk group under different clinical parameter stratification was worse than that in the low-risk group (Figure 5), suggesting that our risk score can accurately identify ccRCC patients with poor prognosis under different clinical conditions. These results demonstrated that the seven URGs-based prognostic signature could be used to predict the prognosis of patients with ccRCC regardless of clinical parameters.

### Relationship Between URGs-Based Prognostic Signature and Different Clinical Characteristics

**FIGURE 6:**
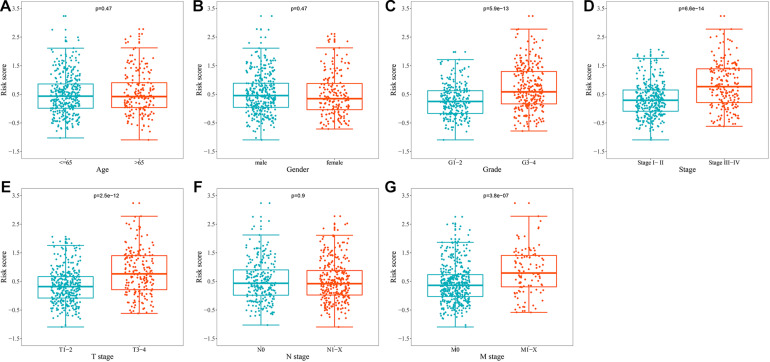
Relationship between the URGs-based prognostic signature and clinical parameters. **(A)** Age (*n* = 539); **(B)** gender (*n* = 539); **(C)** grade (*n* = 531); **(D)** stage (*n* = 536); **(E)** T stage (*n* = 539); **(F)** N stage (*n* = 539); **(G)** M stage (*n* = 537) (the statistical method was *t*-test with only one test).

To explore whether prognostic signature could assess the degree of tumor progression, we compared the differences of risk score for different clinical characteristics. The results indicated that no significant differences were observed in risk scores between groups after stratification by age, gender, and N stage (Figures 6A,B,F). However, the risk score of tumor grade 3–4 was significantly higher than that of tumor grade 1–2 (*P* = 5.9e-13, Figure 6C), the risk score of tumor stage III-IV was significantly higher than that of tumor stage I-II (*P* = 6.6e-14, Figure 6D), the risk score of tumor T stage 3–4 was significantly higher than that of tumor T stage 1–2 (*P* = 2.5e-12, Figure 6E), and the risk score of tumor M stage 1-X was significantly higher than that of tumor M stage 0 (*P* = 3.8e-07, Figure 6G). These results suggested that prognostic signature can be used to assess the degree of progression of ccRCC tumors, and the higher the risk score, the higher the malignant degree of tumors.

### Assessment of the Association Between Prognostic URGs and Different Clinical Characteristics

In addition to the above analysis, we also preliminarily explored the possible roles of these seven URGs in ccRCC according to different clinical characteristics. We stratified the expression levels of these URGs based on different clinical variables, and then compared the differences in expression levels between the two groups. The results indicated that CDCA3, CHFR, CORO6, RNF175, TRIM72, VAV3, and WDR72 were significantly correlated with grade, stage, and T stage (Table 2); the correlation between CDCA3, CHFR, CORO6, RNF175, VAV3, and WDR72 and M stage was statistically significant (Table 2). However, no genes differed significantly with gender, or N stage (Table 2). These results suggested that these prognostic URGs may play an important role in the tumor progression of ccRCC, which is worthy of further study.

**TABLE 2:** The relationship between prognostic related ubiquitin genes and clinicopathologic parameters.

**Gene**		**Gender (male/female)**	**Grade (G1–2/G3–4)**	**Stage (I–II/III–IV)**	**T stage (T1–T2/T3–T4)**	**N stage (N0/N1-X)**	**M stage (M0/M1-X)**
N		353/186	249/282	331/205	349/190	241/298	428/109
CDCA3	*t*-value	1.687	NA*	NA*	NA*	0.519	NA*
	*P*-value	0.092	<0.001	<0.001	<0.001	0.604	<0.001
CHFR	*t*-value	0.073	NA*	5.967	5.670	0.139	4.574
	*P*-value	0.942	<0.001	<0.001	<0.001	0.890	<0.001
CORO6	*t*-value	1.650	3.427	3.504	3.454	1.014	4.538
	*P*-value	0.100	<0.001	<0.001	<0.001	0.311	<0.001
RNF175	*t*-value	1.331	3.750	4.174	4.112	0.050	2.502
	*P*-value	0.184	<0.001	<0.001	<0.001	0.960	0.013
TRIM72	*t*-value	NA*	2.168	2.548	2.048	NA*	NA*
	*P*-value	0.373	0.031	0.011	0.041	0.888	0.086
VAV3	*t*-value	NA*	NA*	NA*	NA*	0.815	NA*
	*P*-value	0.323	<0.001	<0.001	<0.001	0.416	<0.001
WDR72	*t*-value	1.774	NA*	NA*	NA*	0.289	NA*
	*P*-value	0.077	<0.001	<0.001	<0.001	0.773	<0.001

NA, not available. *Non-parametric Mann-Whitney rank sum test. The statistical method was *t*-test or non-parametric Mann-Whitney rank sum test with only one test.

### Multidimensional Regulatory Network of Prognostic URGs and Functional Enrichment Analysis

**FIGURE 7:**
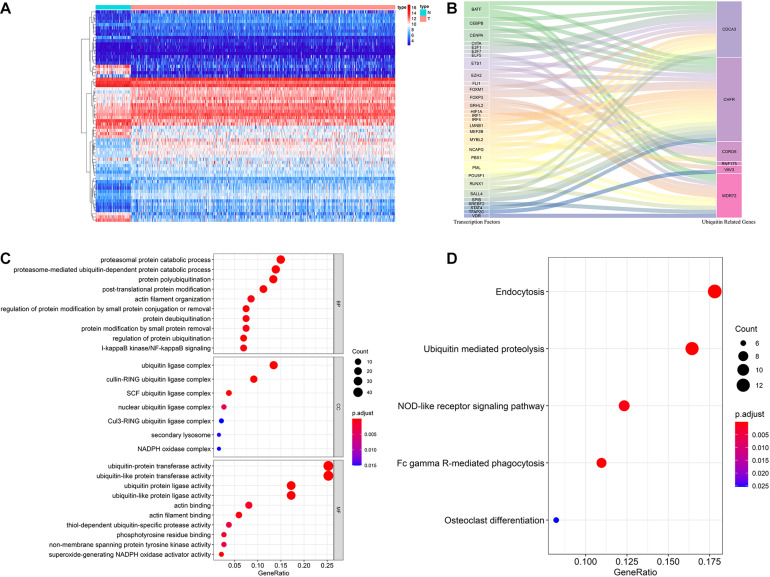
Multidimensional regulatory network of prognostic URGs and functional enrichment analysis. **(A)** The differential expression of 66 TFs in ccRCC tissue samples (*n* = 539) compared with normal renal samples (*n* = 72) is shown in the heatmap; **(B)** Sankey plot of URGs-TFs regulatory networks; **(C)** GO enrichment analysis of the differentially expressed URGs; **(D)** KEGG enrichment analysis of the differentially expressed URGs (the statistical method was multiple hypothesis testing).

The ubiquitin-proteasome system plays an important regulatory role in the general transcription process, and through this role affects the function and activity of TFs. Cui et al. (2017) found that hypoxia enhanced the stability and transcriptional activity of HIF-1α through SENP1, thereby enhancing the stemness of hepatocellular carcinoma cells and hepatocarcinogenesis. Jin et al. (2017) found that FBW7 inhibits invasion of pancreatic cancer cells by inhibiting EZH2 activity and degrading EZH2. Therefore, it is worthwhile to reveal the regulatory networks of prognostic URGs and TFs in tumor genesis and progression. In our study, we downloaded 318 TFs from the Cistrome Project, extracted 314 ccRCC-related TFs based on the TCGA cohort, and finally obtained 66 differentially expressed TFs, including 46 up-regulated and 20 down-regulated TFs. The expression heatmap of these TFs is shown in Figure 7A. By co-expression analysis of differentially expressed TFs and prognostic URGs, a total of 54 TFs involved in regulation were identified. The regulatory network of the URGs-TFs is shown in Figure 7B, in which 10 TFs negatively regulated corresponding URGs and 44 TFs positively regulated them. The specific regulation relationship between them is shown in Supplementary Table 2.

In order to investigate the molecular functions and biological pathways of these differentially expressed URGs, the “clusterProfiler” package was used to perform GO and KEGG enrichment analysis on these URGs. Biological process analysis showed that these URGs were mainly concentrated in protein polyubiquitination, proteasomal protein catabolic process, proteasome-mediated ubiquitin-dependent protein catabolic process, post-translational protein modification, protein deubiquitination, I-kappaB kinase/NF-kappaB signaling, and regulation of protein ubiquitination (Figure 7C). Cellular component analysis showed that these URGs were mainly concentrated in ubiquitin ligase complex, cullin-RING ubiquitin ligase complex, and SCF ubiquitin ligase complex (Figure 7C). Molecular function analysis showed that these URGs were mainly concentrated in ubiquitin-protein transferase activity, ubiquitin-like protein transferase activity, ubiquitin protein ligase activity, phosphotyrosine residue binding, and superoxide-generating NADPH oxidase activity (Figure 7C). In terms of KEGG analysis, these differentially expressed URGs were mainly concentrated in Ubiquitin mediated proteolysis, Fc gamma R-mediated phagocytosis, NOD-like receptor signaling pathway, and Osteoclast differentiation (Figure 7D).

### Evaluation of the Relationship Between the Prognostic Signature and the Degree of Immune Cell Infiltration

**FIGURE 8:**
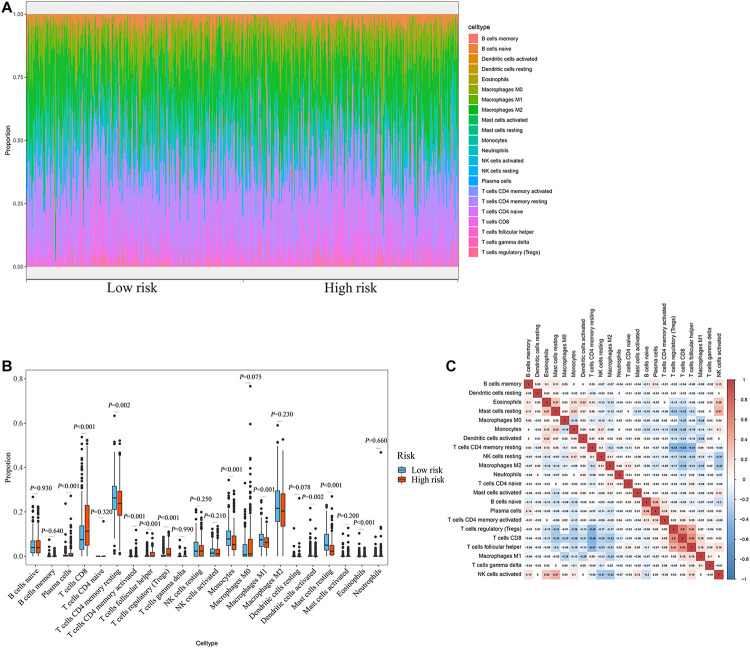
Relationship between prognostic signature and immune cell infiltration. **(A)** Stacked bar chart of the distribution of 22 immune cells in each ccRCC sample of the TCGA cohort (*n* = 539). **(B)** Box plot of immune cell infiltrates in ccRCC patients at high- (*n* = 269) and low-risk (270). **(C)** Immune cell proportional correlation matrix (the statistical method was *t*-test).

The degree of immune cell infiltration affects tumor progression and therapeutic effect. In this study, we evaluated the differences in immune cell infiltration between different subgroups based on the CIBERSORT algorithm. The results showed significant differences in the composition of the 22 immune cells in each sample in the TCGA cohort (Figure 8A). Specifically, the infiltration degree of plasma cells, T cells CD8, T cells CD4 memory resting, T cells CD4 memory activated, T cells follicular helper, T cells regulatory (Tregs), monocytes, macrophages M1, dendritic cells activated, mast cells resting, and eosinophils were significantly different between the high- and low-risk groups (Figure 8B), suggesting that there may be differences in immune status between the high- and low-risk groups. Correlation matrix results revealed that the T cells CD8 had the strongest positive correlation with T cells regulatory (Tregs), was also positively correlated with T cells follicular helper (Figure 8C).

### Evaluation of the Prognostic Significance of Different Clinical Characteristics in ccRCC Patients and Construction of a Nomogram

**FIGURE 9:**
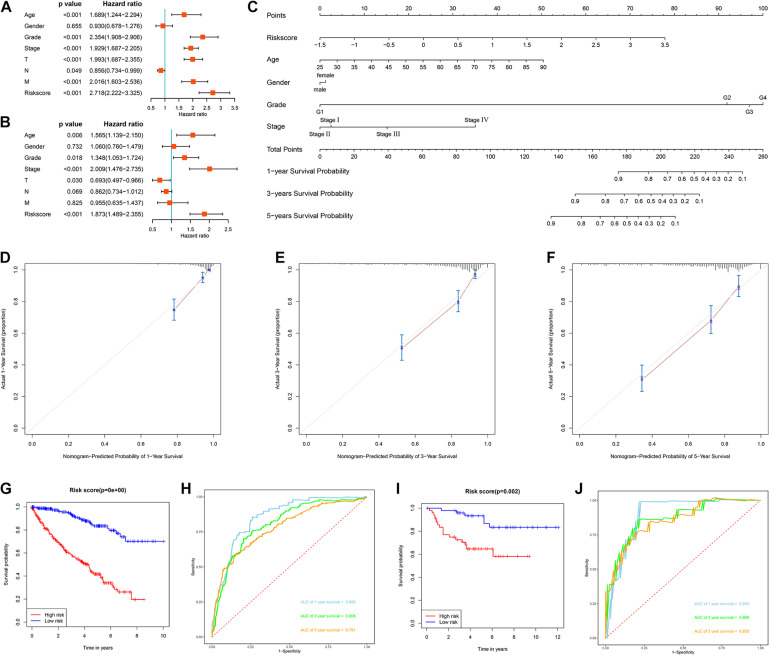
Evaluation of the prognostic significance of different clinical characteristics in ccRCC patients and construction of a nomogram. **(A)** Univariate Cox regression analyses in the TCGA cohort ccRCC patients (*n* = 539); **(B)** multivariate Cox regression analyses in the TCGA cohort ccRCC patients (*n* = 526); **(C)** the nomogram for predicting the 1-, 3-, and 5-year OS of ccRCC patients (*n* = 526); **(D)** the calibration curve of the nomogram for predicting 1-year OS of ccRCC patients; **(E)** the calibration curve of the nomogram for predicting 3-year OS of ccRCC patients; **(F)** the calibration curve of the nomogram for predicting 5-year OS of ccRCC patients; **(G)** Kaplan-Meier survival curve analysis in the TCGA cohort based the nomogram (*n* = 526); **(H)** ROC curve analysis shows 1, 3, and 5-year OS and the corresponding AUC values for ccRCC patients from the TCGA cohort based the nomogram (*n* = 526); **(I)** Kaplan-Meier survival curve analysis in the E-MTAB-1980 cohort based the nomogram (*n* = 99); **(J)** ROC curve analysis shows 1, 3, and 5-year OS and the corresponding AUC values for ccRCC patients from the E-MTAB-1980 cohort based the nomogram (*n* = 99) (the statistical method was a log-rank test for a single factor).

We first evaluated the prognostic value of different clinical characteristics in patients with ccRCC through univariate Cox proportional hazards regression analysis. The results showed that the age (*P* < 0.001), tumor grade (*P* < 0.001), tumor stage (*P* < 0.001), primary tumor location (*P* < 0.001), lymph node infiltration (*P* = 0.049), distant metastasis (*P* < 0.001), and risk score (*P* < 0.001) of ccRCC patients were significantly correlated with OS (Figure 9A). However, multivariate Cox proportional hazards regression analysis revealed that age (*P* = 0.006), tumor grade (*P* = 0.018), tumor stage (*P* < 0.001), primary tumor location (*P* = 0.030), and risk score (*P* < 0.001) affected OS as independent prognostic factors (Figure 9B).

Next, based on these seven prognostic URGs, we established a nomogram that could quantitatively predict the prognosis of patients with ccRCC (Figure 9C). Briefly, the points of each variable were mapped to the corresponding horizontal line, then the total points of each patient were calculated and normalized to a distribution of 0–100. This allows us to estimate 1-, 3-, and 5-year survival rates for ccRCC patients based on the prognosis axis and total point axis, which can be used as a reference for clinical decision-making. The results of the calibration curve at different time points showed that there is a strong consistency between the predicted value of the nomogram and the actual value (Figures 9D–F). Additionally, we further evaluated the clinical applicability and validity of the nomogram using the TCGA and E-MTAB-1980 cohorts. Survival analysis using Kaplan-Meier method showed that nomogram can accurately identify ccRCC patients with low survival probability in the TCGA and E-MTAB-1980 cohorts (*P* < 0.001 and *P* = 0.002, Figures 9G,I). Based on the nomogram, in the TCGA cohort, the predicted AUCs were 0.856 at 1 year, 0.806 at 3 years, and 0.781 at 5 years (Figure 9H), and in the E-MTAB-1980 dataset, the predicted AUCs were 0.893 at 1 year, 0.868 at 3 years, and 0.855 at 5 years (Figure 9J), indicating that the nomogram had good predictive power and accuracy.

### IHC Staining Analysis

**FIGURE 10:**
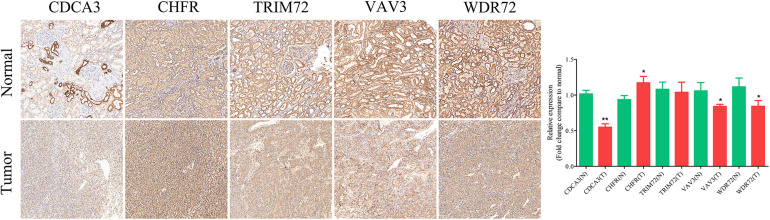
Validation of the expression of the prognostic URGs in ccRCC and normal renal tissues by immunohistochemical staining analysis. The expressions of CDCA3, CHFR, TRIM72, VAV3, and WDR72 in ccRCC tissues and adjacent non-tumor renal tissues were detected by immunohistochemical staining (magnification 100×). Quantification of immunohistochemical staining for CDCA3, CHFR, TRIM72, VAV3, and WDR72 by Image Pro Plus software. *P < 0.05 vs. normal group, **P < 0.01 vs. normal group.

IHC assay was used to preliminarily verify the protein expression levels of these URGs between normal kidney tissues and ccRCC tissues. The results revealed that CDCA3 (*P* < 0.001), VAV3 (*P* = 0.034), and WDR72 (*P* = 0.033) were low expressed in ccRCC tissues compared with normal renal tissues. However, the CHFR (*P* = 0.018) were high expressed in ccRCC tissues compared with normal renal tissues (Figure 10). All the results of IHC analysis were shown in Supplementary Table 3.

## Discussion

Ubiquitin modification is a PTM of proteins in pathophysiological processes that plays a regulatory role in complex biological processes, including protein-protein interactions, protein activation, and substrate activation or inactivation (Pickart and Eddins, 2004). Abnormalities in the ubiquitin-modifying system are responsible for a variety of diseases, including neurodegenerative diseases, autoimmune diseases, and tumors. Abnormal expression of E3S and DUBS has been found to affect human malignancies by regulating the activity of tumor-associated proteins (Love et al., 2013; Paul et al., 2017). However, only a small number of ubiquitin molecules have been thoroughly studied, and most of the research has focused on the function of individual genes. Few studies have systematically explored the molecular characteristics and prognostic potential of URGs using expression profile datasets. In our study, we identified 204 differentially expressed URGs, including 141 up-regulated URGs and 63 down-regulated URGs. The biological functions and molecular mechanisms of these URGs were systematically analyzed by using bioinformatics techniques. A total of seven prognostic related URGs were identified by Cox regression analysis of differential URGs and used to construct a prognostic signature. We also analyzed the correlation between prognostic signature, prognostic URGs and clinical characteristics. Additionally, we further revealed the regulatory network of URGs-TFs and the relationship between prognostic signature and immune cell infiltration.

Through the Cox proportional hazards regression analysis of URGs, we screened out a total of seven URGs including *CDCA3*, *CHFR*, *CORO6*, *RNF175*, *TRIM72*, *VAV3*, and *WDR72*. CDCA3 is a major regulator of mitosis and cell cycle. CDCA3 overexpression has been reported to promote the G1/S phase transformation and promote the proliferation of colorectal cancer cells by activating the NF-kB/cyclin D1 signaling pathway (Zhang et al., 2018). Liu et al. (2020) found that in RCC, the long non-coding RNA SNHG12 promoted tumor progression and sunitinib resistance by upregulating CDCA3. CHFR plays an important role in cell cycle regulation. Numerous studies have shown that the CHFR gene is significantly silenced or mutated by promoter methylation in many cancer types including non-small cell lung cancer (Mizuno et al., 2002) and esophageal cancer (Shibata et al., 2002). Yang et al. (2019) found that CHFR promoted the invasion of gastric cancer cells by inducing epithelial to mesenchymal transformation in a HDAC1-dependent manner. Coronin-6, a gene product of CORO6, is a member of the coronin family and has been shown to play a role in cell movement, vesicle transport, and cell division (Roadcap et al., 2008). Studies have shown that CORO6 is a potential tumor suppressor in renal cancer (Morris et al., 2011). Kiely et al. (2020) found that low CORO6 expression was associated with poorer overall breast cancer survival. RNF175 and RNF213 share their E3 ubiquitin ligase activity and play an important role in protein post-translational ubiquitination modification (Kaneko et al., 2016). TRIM72 is a member of the tripartite motif family. Studies suggest that TRIM72 ubiquitin ligase activity may be associated with insulin resistance and metabolic syndrome, a well-known risk factor for colon cancer (Liu et al., 2018). Fernández-Aceñero et al. (2020) found that immunohistochemical expression of TRIM72 could predict colorectal cancer recurrence. VAV3 is a member of the guanine nucleotide exchange factor family and is involved in many important pathological processes, including tumorigenesis and cell transformation. Studies have shown that VAV3 expression is increased in a variety of cancers and can promote gastric cancer cell metastasis (Aguilar et al., 2014; Xie et al., 2019). The WDR72 gene encodes proteins that promote the formation of heterotrimeric or multiprotein complexes. WDR proteins may act as molecular adapters for substrate recognition and regulate a variety of biological processes through ubiquitin-independent proteolysis. Mares et al. (2013) found that WDR72 can be used as a biomarker for predicting low- and moderate-risk recurrence of non-muscularly invasive bladder cancer. These results suggested that these URGs play important roles in a variety of tumors and may be involved in the occurrence and development of ccRCC. However, further experiments *in vitro* and *in vivo* are needed to explore the exact molecular mechanisms of these URGs.

Subsequently, we developed a URGs-based prognostic signature for OS. Survival analysis by Kaplan-Meier method showed that patients in the high-risk group had a shorter OS than those in the low-risk group. ROC curve analysis showed that the URGs-based prognostic signature could better screen ccRCC patients with poor prognosis. Further analysis showed that the prognosis of patients in each high-risk group under different clinical parameter stratification was worse than that in the low-risk group. Moreover, we also found that the prognostic signature can be used to assess the degree of progression of ccRCC tumors. These results suggested that this prognostic signature has a good ability to distinguish the degree of malignancy and prognosis of ccRCC patients.

Moreover, we explored the URGs-TFs regulatory network based on the TCGA cohort, and co-expression analysis revealed a regulatory network consisting of 6 prognostic URGs and 54 differentially expressed TFs. The function and activity of these TFs may be affected, thereby regulating the occurrence and progression of tumors, which is worthy of further study. Subsequent GO and KEGG enrichment analysis indicated that these differentially expressed URGs were mainly concentrated in protein polyubiquitination, proteasomal protein catabolic process, proteasome-mediated ubiquitin-dependent protein catabolic process, post-translational protein modification, protein deubiquitination, regulation of protein ubiquitination, and ubiquitin mediated proteolysis. Ubiquitination has a wide range of cellular functions, including proteolytic and non-proteolytic effects, such as proteasomal degradation of proteins, internalization and down-regulation of receptors, assembly of multi-protein complexes, inflammatory signaling, autophagy, DNA repair, and regulation of enzyme activity (Grabbe et al., 2011). Thus, dysregulation of ubiquitination can have a wide range of effects. It may cause abnormal activation or deactivation ways (such as those involved in tumor formation, or cell metabolism), inappropriate or inadequate protein complex assembly (such as occurred in the process of regulating inflammation or DNA repair), or the accumulation of misfolded proteins (in neurodegenerative diseases during endoplasmic reticulum or in the cytoplasm) (Hoeller and Dikic, 2009; Popovic et al., 2014). Additionally, ubiquitination also regulates T cell development, activation, and differentiation, thereby mediating and maintaining effective adaptive immune responses and immune tolerance. Dysregulated events of ubiquitination are associated with immune disorders including autoimmune diseases and inflammatory diseases (Hu and Sun, 2016). Further studies found significant differences in the degree of immune cell infiltration between the high-risk and low-risk groups according to the prognostic signature. These results suggested that ubiquitination and its dysregulation may affect the occurrence and development of tumors through a variety of pathways.

Overall, this study provides a new insight into the tumorigenesis and progression of ccRCC from the perspective of ubiquitin. These seven URGs-based prognostic signature has a better effect on the prediction of survival in ccRCC patients. In addition, URGs-based prognostic signature show important biological functions and clinical value, suggesting that they may be used in adjuvant clinical therapy. However, our study also has some limitations. First of all, the construction and validation of this signature is based on retrospective analysis, and prospective clinical cohort validation is also required. Secondly, different platforms may lead to differences in patients due to their heterogeneity. Finally, the specific functions and molecular mechanisms of these prognostic URGs in ccRCC are still unclear, and this study may also omit some URGs that have significant influence on disease progression but are rarely expressed, which require follow-up attention and further experimental exploration.

## Conclusion

In summary, through multiple bioinformatics analyses, we systematically explored the molecular characteristics and prognostic value of URGs in ccRCC based on the high-throughput sequencing expression profile datasets, and preliminarily revealed the complex biological functions and immune processes involved in these molecules and their regulatory networks. These URGs may be involved in the occurrence, development, invasion, and metastasis of ccRCC. We also constructed a prognostic signature that could independently predict prognosis in ccRCC patients. Our results will help to reveal the pathogenesis of ccRCC and develop new biomarkers, and provide certain guiding significance for clinical decision-making.

## Data Availability Statement

The data and materials can be obtained by contacting the corresponding author.

## Ethics Statement

The studies involving human tissues samples were reviewed and approved by the Research Ethics Committee of Tongji Hospital, Tongji Medical College, Huazhong University of Science and Technology, and complied with the Declaration of Helsinki. All patients were aware of the present study and signed an informed consent agreement.

## Author Contributions

YW designed the study and performed the data analysis. XZ carried out the immunohistochemical experiments, performed the data analysis, and revised the manuscript. XW, HF, BH, ZD, and BL performed the data analysis. YL, YR, XL, ZL, and JL performed the data analysis and revised the manuscript. TW designed the study and revised the manuscript. All authors read and approved the final manuscript.

## Conflict of Interest

The authors declare that the research was conducted in the absence of any commercial or financial relationships that could be construed as a potential conflict of interest.

## Footnotes

1 https://portal.gdc.cancer.gov/

2 http://iuucd.biocuckoo.org/index.php

3 http://www.bioconductor.org/packages/release/bioc/html/edgeR.html

4 https://www.ebi.ac.uk/arrayexpress/experiments/E-MTAB-1980/

5 www.cistrome.org

6 http://www.bioconductor.org/packages/release/bioc/html/clusterProfiler.html

## Abbreviations

RCC: Renal cell carcinoma
CcRCC: Clear cell renal cell carcinoma
TCGA: The Cancer Genome Atlas
PTM: Post-translational modification
E1s: Ubiquitin-activating enzymes
E2s: Ubiquitin-conjugating enzymes
E3s: Ubiquitin protein ligases
UBD: Ubiquitin-binding domain-containing protein
ULDs: Ubiquitin-like domains
DUBs: deubiquitinases
URGs: Ubiquitin related genes
OS: Overall survival
FC: Fold change
FDR: False discovery rate
LASSO: Least absolute shrinkage and selection operator
ROC: Receiver operating characteristic
TFs: Transcription factors
GO: Gene ontology
KEGG: Kyoto Encyclopedia of Genes and Genomes
AUC: Area under the receiver operating characteristic curve
CIBERSORT: Cell type identification by estimating relative subsets of RNA transcripts
IHC: Immunohistochemical.
