# Supplementary material for: Development of an Individualized Ubiquitin Prognostic Signature for Clear Cell Renal Cell Carcinoma
Source: Front Cell Dev Biol. 2021 Jun 22;9:684643. doi: 10.3389/fcell.2021.684643 (PMC8258262; doi:10.3389/fcell.2021.684643)
Supplement: Supplementary file 1 [file Data_Sheet_1.docx]

**Development of an individualized ubiquitin prognostic signature for clear cell renal cell carcinoma**

**Table S1**. Univariate Cox regression analysis of differentially expressed URGs.

| Gene | Hazard ratio | P value | Gene | Hazard ratio | P value |
| --- | --- | --- | --- | --- | --- |
| AIRE | 1.258 | <0.001 | PDZRN3 | 0.828 | <0.001 |
| ANKRD13B | 1.454 | <0.001 | PDZRN4 | 0.903 | 0.014 |
| AP1G2 | 1.435 | <0.001 | PELI2 | 0.653 | <0.001 |
| ARHGAP26 | 0.792 | 0.011 | PML | 2.218 | <0.001 |
| ARHGAP4 | 1.653 | <0.001 | PPARA | 0.553 | <0.001 |
| ARPC1B | 1.665 | <0.001 | PSTPIP1 | 1.271 | <0.001 |
| ASAP2 | 0.748 | <0.001 | RAB40AL | 1.394 | <0.001 |
| ATG12 | 1.601 | 0.016 | RASD2 | 1.186 | 0.021 |
| ATG16L2 | 1.295 | <0.001 | RBCK1 | 1.682 | <0.001 |
| BAG1 | 0.548 | <0.001 | RNF113B | 1.150 | 0.004 |
| BAZ1A | 1.685 | <0.001 | RNF149 | 1.747 | <0.001 |
| BCL6B | 0.807 | 0.001 | RNF151 | 1.316 | 0.001 |
| BRSK2 | 1.160 | 0.003 | RNF152 | 0.706 | <0.001 |
| BTBD11 | 1.197 | <0.001 | RNF166 | 2.005 | <0.001 |
| BTBD19 | 1.393 | <0.001 | RNF175 | 1.403 | <0.001 |
| CCNF | 1.895 | <0.001 | RNF222 | 1.238 | 0.001 |
| CDC20 | 1.521 | <0.001 | RNF43 | 0.805 | <0.001 |
| CDCA3 | 1.707 | <0.001 | SACS | 1.516 | 0.001 |
| CHFR | 2.850 | <0.001 | SEC31B | 1.298 | <0.001 |
| CORO1A | 1.238 | 0.004 | SH3D21 | 1.364 | <0.001 |
| CORO2B | 1.158 | 0.005 | SH3GL1 | 1.401 | 0.011 |
| CORO6 | 1.348 | <0.001 | SH3GL3 | 1.276 | <0.001 |
| CORO7 | 1.586 | <0.001 | SPSB1 | 1.233 | 0.001 |
| DCAF11 | 0.484 | <0.001 | STAC3 | 1.782 | <0.001 |
| DCAF4L2 | 1.179 | 0.001 | TAF1D | 1.478 | 0.002 |
| DNAJC6 | 0.801 | 0.003 | TBL1Y | 0.902 | 0.020 |
| DPF1 | 1.428 | <0.001 | THOC6 | 1.702 | <0.001 |
| DTX1 | 1.312 | <0.001 | TNIP1 | 0.693 | 0.002 |
| DTX2 | 1.879 | <0.001 | TNIP3 | 1.169 | <0.001 |
| EML5 | 0.857 | 0.002 | TNK2 | 1.467 | <0.001 |
| EPN3 | 1.082 | 0.013 | TOM1L2 | 0.685 | 0.009 |
| FBXL6 | 1.781 | <0.001 | TRAF2 | 1.677 | <0.001 |
| FBXO6 | 1.377 | 0.001 | TRIM15 | 0.941 | 0.049 |
| FCHSD1 | 1.483 | <0.001 | TRIM2 | 0.609 | <0.001 |
| GNB3 | 1.299 | <0.001 | TRIM36 | 1.433 | <0.001 |
| HECW2 | 0.764 | <0.001 | TRIM46 | 1.348 | <0.001 |
| ISG15 | 1.360 | <0.001 | TRIM54 | 1.062 | 0.030 |
| KCTD13 | 1.845 | <0.001 | TRIM55 | 0.860 | <0.001 |
| KLHL13 | 0.763 | 0.001 | TRIM59 | 1.525 | 0.001 |
| KLHL17 | 1.454 | <0.001 | TRIM72 | 1.243 | <0.001 |
| KLHL33 | 0.880 | 0.032 | UBE2C | 1.437 | <0.001 |
| KLHL35 | 1.191 | <0.001 | UBL4B | 1.244 | <0.001 |
| LAPTM5 | 1.198 | 0.030 | UBXN11 | 1.307 | 0.001 |
| LLGL2 | 0.825 | 0.020 | UCHL1 | 1.149 | <0.001 |
| LNX1 | 0.745 | <0.001 | UHRF1 | 1.496 | <0.001 |
| LONRF2 | 1.085 | 0.036 | USP2 | 0.814 | <0.001 |
| MYO1F | 1.332 | 0.001 | USP53 | 0.548 | <0.001 |
| NCF4 | 1.472 | <0.001 | VAV1 | 1.182 | 0.037 |
| NEB | 1.121 | 0.001 | VAV3 | 0.658 | <0.001 |
| NEBL | 0.752 | <0.001 | WDR27 | 1.338 | <0.001 |
| NEDD4L | 0.705 | <0.001 | WDR38 | 1.138 | 0.032 |
| NEURL1B | 0.800 | 0.006 | WDR49 | 1.187 | 0.006 |
| NOD2 | 1.404 | <0.001 | WDR62 | 1.629 | <0.001 |
| NTNG2 | 1.282 | <0.001 | WDR72 | 0.774 | <0.001 |
| OASL | 1.349 | <0.001 | WDR90 | 1.341 | 0.002 |
| PCGF1 | 1.681 | 0.001 | WSB1 | 1.438 | <0.001 |
| PCGF3 | 1.639 | 0.001 | ZBTB8B | 1.180 | 0.006 |


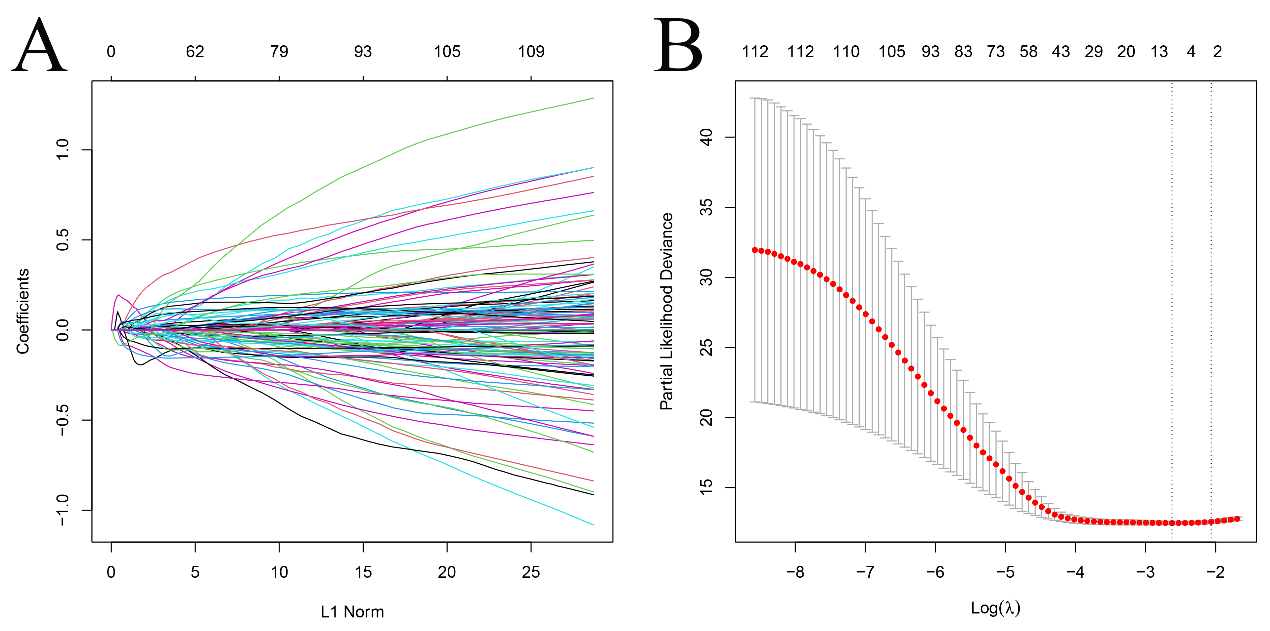


**Figure S1**. LASSO regression analysis for screening prognosis related URGs.

(A) Trajectories of model coefficients; (B) Cross validation fitting and performance

evaluation of the model.

**Table S2**. Regulatory relationships between these TFs and prognostic related URGs.

| Transcription factors | Ubiquitin genes | Coefficient | P value | Regulation |
| --- | --- | --- | --- | --- |
| PML | CDCA3 | 0.336 | 1.06E-15 | Positive |
| EZH2 | CDCA3 | 0.637 | 1.43E-62 | Positive |
| E2F1 | CDCA3 | 0.676 | 4.06E-73 | Positive |
| BATF | CDCA3 | 0.343 | 2.53E-16 | Positive |
| FOXM1 | CDCA3 | 0.813 | 5.77E-128 | Positive |
| NCAPG | CDCA3 | 0.818 | 5.81E-131 | Positive |
| CENPA | CDCA3 | 0.893 | 6.54E-188 | Positive |
| RUNX1 | CDCA3 | 0.341 | 4.14E-16 | Positive |
| LMNB1 | CDCA3 | 0.698 | 6.74E-80 | Positive |
| MYBL2 | CDCA3 | 0.821 | 6.57E-133 | Positive |
| ETS1 | CDCA3 | -0.328 | 5.50E-15 | Negative |
| E2F7 | CDCA3 | 0.731 | 3.29E-91 | Positive |
| CEBPB | CDCA3 | 0.355 | 2.02E-17 | Positive |
| SALL4 | CDCA3 | 0.514 | 9.33E-38 | Positive |
| PML | CHFR | 0.421 | 1.27E-24 | Positive |
| EZH2 | CHFR | 0.567 | 2.90E-47 | Positive |
| POU5F1 | CHFR | 0.397 | 7.56E-22 | Positive |
| STAT4 | CHFR | 0.504 | 4.30E-36 | Positive |
| BATF | CHFR | 0.498 | 3.55E-35 | Positive |
| PBX1 | CHFR | -0.376 | 1.37E-19 | Negative |
| FOXM1 | CHFR | 0.302 | 7.37E-13 | Positive |
| NCAPG | CHFR | 0.303 | 6.27E-13 | Positive |
| CENPA | CHFR | 0.370 | 5.63E-19 | Positive |
| RUNX1 | CHFR | 0.373 | 3.01E-19 | Positive |
| LMNB1 | CHFR | 0.350 | 5.58E-17 | Positive |
| MEF2B | CHFR | 0.583 | 1.80E-50 | Positive |
| MYBL2 | CHFR | 0.375 | 2.11E-19 | Positive |
| ETS1 | CHFR | -0.312 | 1.29E-13 | Negative |
| CIITA | CHFR | 0.404 | 1.58E-22 | Positive |
| IRF1 | CHFR | 0.303 | 6.21E-13 | Positive |
| CEBPB | CHFR | 0.320 | 2.91E-14 | Positive |
| IRF4 | CHFR | 0.313 | 9.71E-14 | Positive |
| FOXP3 | CHFR | 0.474 | 1.36E-31 | Positive |
| SALL4 | CHFR | 0.361 | 5.25E-18 | Positive |
| SPIB | CHFR | 0.302 | 8.62E-13 | Positive |
| EZH2 | CORO6 | 0.352 | 3.96E-17 | Positive |
| FLI1 | CORO6 | -0.316 | 5.54E-14 | Negative |
| CENPA | CORO6 | 0.374 | 2.67E-19 | Positive |
| MYBL2 | CORO6 | 0.419 | 2.32E-24 | Positive |
| ETS1 | CORO6 | -0.364 | 2.57E-18 | Negative |
| BATF | RNF175 | 0.370 | 6.03E-19 | Positive |
| GRHL2 | VAV3 | 0.351 | 4.63E-17 | Positive |
| TFAP2C | VAV3 | 0.301 | 9.47E-13 | Positive |
| ELF5 | WDR72 | 0.303 | 7.06E-13 | Positive |
| SREBF2 | WDR72 | 0.462 | 8.23E-30 | Positive |
| PML | WDR72 | -0.479 | 2.47E-32 | Negative |
| HIF1A | WDR72 | 0.321 | 2.18E-14 | Positive |
| VDR | WDR72 | 0.417 | 4.07E-24 | Positive |
| BATF | WDR72 | -0.314 | 8.31E-14 | Negative |
| GRHL2 | WDR72 | 0.359 | 8.05E-18 | Positive |
| PBX1 | WDR72 | 0.312 | 1.24E-13 | Positive |
| RUNX1 | WDR72 | -0.446 | 1.19E-27 | Negative |
| CEBPB | WDR72 | -0.318 | 3.71E-14 | Negative |
| FOXP3 | WDR72 | -0.318 | 4.06E-14 | Negative |

Table S3. Relative expression levels of URGs in ccRCC tissues and normal kidney tissues.

|  | Normal | | | Tumor | | | t | p |
| --- | --- | --- | --- | --- | --- | --- | --- | --- |
| CDCA3 | 1.000 | 1.072 | 0.974 | 0.548 | 0.503 | 0.597 | 11.650 | <0.001 |
| CHFR | 1.000 | 0.916 | 0.893 | 1.138 | 1.273 | 1.105 | 3.876 | 0.018 |
| TRIM72 | 1.000 | 1.049 | 1.194 | 1.014 | 0.910 | 1.191 | 0.420 | 0.696 |
| VAV3 | 1.000 | 1.192 | 0.983 | 0.830 | 0.874 | 0.816 | 3.155 | 0.034 |
| WDR72 | 1.000 | 1.099 | 1.246 | 0.845 | 0.921 | 0.758 | 3.194 | 0.033 |
